# Acoustic-level and language-specific processing of native and non-native phonological sequence onsets in the low gamma and theta-frequency bands

**DOI:** 10.1038/s41598-021-03611-2

**Published:** 2022-01-10

**Authors:** Monica Wagner, Silvia Ortiz-Mantilla, Mateusz Rusiniak, April A. Benasich, Valerie L. Shafer, Mitchell Steinschneider

**Affiliations:** 1 St. John’s University, St. John’s Hall, Room 344 e1, 8000 Utopia Parkway, Queens, NY 11439 USA; 2 Rutgers University, Newark, NJ 07102 USA; 3 BESA, GmbH, 82166 Gräfelfing, Germany; 4 The Graduate Center of the City University of New York, New York, NY 10016 USA; 5 Albert Einstein College of Medicine, Bronx, NY 10461 USA

**Keywords:** Cortex, Human behaviour

## Abstract

Acoustic structures associated with native-language phonological sequences are enhanced within auditory pathways for perception, although the underlying mechanisms are not well understood. To elucidate processes that facilitate perception, time–frequency (T–F) analyses of EEGs obtained from native speakers of English and Polish were conducted. Participants listened to same and different nonword pairs within counterbalanced attend and passive conditions. Nonwords contained the onsets /pt/, /pət/, /st/, and /sət/ that occur in both the Polish and English languages with the exception of /pt/, which never occurs in the English language in word onset. Measures of spectral power and inter-trial phase locking (ITPL) in the low gamma (LG) and theta-frequency bands were analyzed from two bilateral, auditory source-level channels, created through source localization modeling. Results revealed significantly larger spectral power in LG for the English listeners to the unfamiliar /pt/ onsets from the right hemisphere at early cortical stages, during the passive condition. Further, ITPL values revealed distinctive responses in high and low-theta to acoustic characteristics of the onsets, which were modulated by language exposure. These findings, language-specific processing in LG and acoustic-level and language-specific processing in theta, support the view that multi scale temporal processing in the LG and theta-frequency bands facilitates speech perception.

Learning a novel-spoken word requires linking meaning to a word form. Languages allow only a subset of possible phonological sequences within particular prosodic contexts (phonotactics) to constitute word forms. For example, the initial phonological sequence in the word “patina” (i.e., /pə/) is also attested in other English words such as “pastina”, “petunia” and “Patricia”. In contrast, the sequence /pt/ is never found as an initial word sequence in English^1^, but this phonotactic pattern is allowed in other languages, such as Polish (e.g., “ptak” is the Polish word for “bird”)^2,3^. Exposure to language-specific phonotactic patterns during development has been shown to modulate higher-level auditory pathways^4–6^. This suggests that acoustic signals corresponding to native-language phonotactic patterns are somehow enhanced for perception^7–10^, although exactly how requires further study.

Neural activity in the form of oscillations in the electroencephalogram (EEG) can provide insight into how language-specific phonological learning affects word-form processing in the brain. EEG oscillations in the range of high gamma (HG > 70 Hz), low gamma (LG 30–70 Hz), theta (4–8 Hz) and delta (0.5–3 Hz) frequency bands are of special interest for understanding speech processing^11–18^. HG activity in auditory cortex reflects neuronal spiking activity evoked by inputs from lower auditory pathways^19^. A nested sequence of interactions, purportedly, ensues, wherein LG oscillations entrain to acoustic discontinuities at rates corresponding to phonemic-segmental levels of speech, which in turn are modulated by oscillations in lower frequency bands that track the speech envelope at the syllable level (theta) and at the level of words and phrasal units (delta)^12,13,17^. Thus, in this hierarchically nested process of speech decoding, delta activity modulates theta, which in turn regulates activity in the LG and finally HG frequency ranges^19–21^.

Cortical sensory processing of the acoustic aspects of phonemic structures (henceforth termed acoustic-level processing), as well as enhanced language-specific processing of phonemic structures, occurs in the HG frequency band within middle to posterior regions of superior temporal gyrus (STG)^4,15,18,22^. However, whether language-specific phonotactic experience modulates sensory processing in the LG and theta bands remains equivocal^12,20,22,23^.

The rate of change of acoustic signals that correspond to phonemic (~ 25 ms) and syllabic (~ 200 ms) units gives rise to entrainment within corresponding frequency bands, LG (~ 40 Hz) and theta (~ 5 Hz)^16,17,24,25^. It has been conceptualized that speech segmentation within these frequency bands, coordinated hierarchically, supports perception^13,16,20,26,27^. Ghitza, within the model TEMPO, argued that syllable-level tracking in theta, controls higher-frequency phonological-level tracking, in which acoustic signals associated with phonemic transitions are matched to templates in memory^26^. One unknown is whether exposure to a phonotactic pattern in a language modulates LG or theta activations within auditory cortical circuits, which may be interpreted as templates in memory.

To explore hemispheric processing in speech perception, Morillan et al.^22^ examined the electrocorticogram (ECoG) in a native-speaker of French, who had depth electrodes implanted, bilaterally, in three regions of auditory cortex: primary auditory cortex (A1), left posterior temporal plane, right planum temporale (A2), and an anterior region of BA22. The patient listened, passively, to the syllables, “pa” and “ba”. One syllable pair was spoken by an English speaker and one pair was spoken by a French speaker. Evoked responses to the four syllable productions combined were found in LG from left-hemisphere A1, in high-theta (~ 7.5 Hz) from all three cortical regions and in low-theta (~ 5.5 Hz), from the right-hemisphere in planum temporale. Further, high-theta evoked activity to the syllables from the left-hemisphere BA22 was associated with high-amplitude responses in gamma (~ 64 Hz). Thus, analyzing neural responses to phonological sequence onsets in two native-language groups might further clarify acoustic-level and language-specific processing in LG and in high and low-theta frequency sub-bands.

In the present study, high-density EEGs were obtained from 24 native speakers of English and 24 native speakers of Polish (late stage English-language learners) as participants listened to phonological sequence onsets (/pt/, /pət/, /st/ and /sət/) within same and different nonword pairs (Fig. 1). EEGs time-locked to the first word in the pairs were analyzed in the present study. The nonwords within the pairs are phonotactically legal, and therefore possible real words in the English and Polish languages with the exception of words that begin with /pt/, which never occurs in English as a word onset^1^. Each participant was tested on two separate occasions, during counterbalanced (1) attend and (2) passive-listening conditions. For the attend condition, participants performed a syllable identification task to the second word in the word pairs. Alternately, during the passive-listening task, participants were only instructed to listen to the speech stimuli. Henceforth, the passive-listening condition is termed the passive condition.

**Figure 1:**
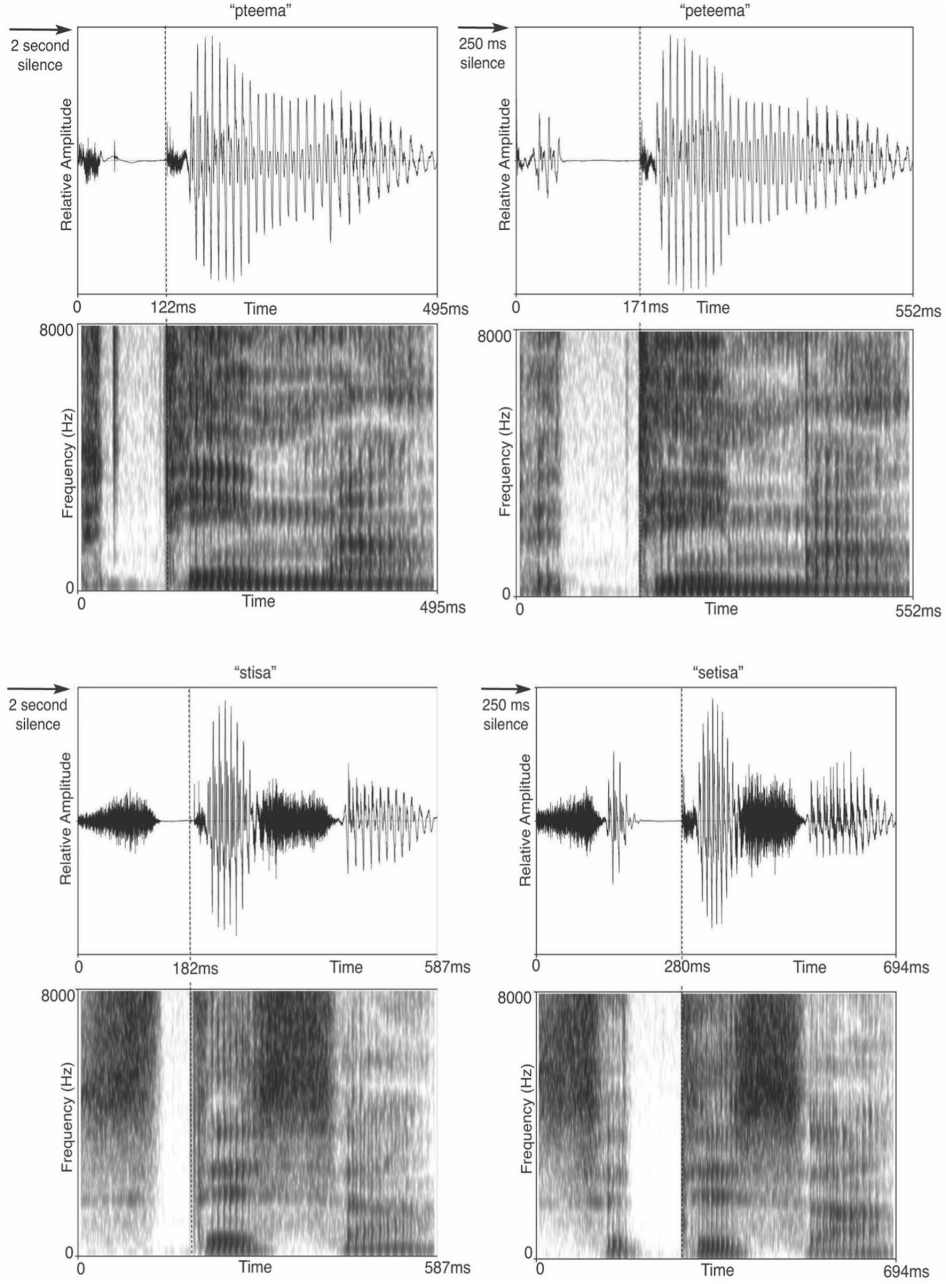
Waveforms/spectrograms illustrating sample stimulus presentation trials. Figure displays the presentation sequence for sample trials. These sample trials illustrate different nonword pairs within the match-to-sample experimental design that highlight phonemic-level processing. Different trials were also presented in the reverse order with three-syllable words preceding two-syllable words. Trials were separated by a fixed two-second interval and nonwords within each pair were separated by a fixed interval of 250 ms. Beneath each waveform is shown the total word duration (far right) and the duration from word onset to the burst for /t/ (vertical dotted line).

The present study addressed the following questions: (1) Do spectral power and/or inter-trial phase locking values differ for Polish compared to English listeners in response to nonwords containing the /pt/ onsets in LG ~ 40 Hz from auditory cortex, and if so, is the response bilateral or restricted to one hemisphere^12,22,26,27^? (2) Alternately, are spectral power and/or inter-trial phase locking values comparable for the Polish and English listeners in response to nonwords containing the /pət/, /st/ and /sət/ onsets in LG ~ 40 Hz from auditory cortex? (3) What are the effects of attention on the neural responses^28–32^? (4) Are modulations that may reflect experience with language-specific phonotactic patterns identified in the theta band, which has been shown to track syllabic-level structures^12,20,23,24^, or are they restricted to the LG frequency band? (5) Is sensory processing of the onsets’ acoustic characteristics differentiated through time–frequency measures in the LG or theta bands^22^?

## Methods

### Participants

Forty-eight adults participated in the study. Half of the participants were native-speakers of English and half were native-speakers of Polish. English speakers were monolingual with parents born in the United States. Bilingual Polish-English speakers came to the US after the age of 15 years with two exceptions. One subject emigrated from Poland at age 10 years and another subject came as a young child and learned English in kindergarten. The mean age of English and Polish participants, recorded at the first testing date, was 27 years (range 21–39) and 30 years (range 21–38 years), respectively. A t-test with alpha set at 0.05, found that the language groups did not differ with respect to age. Participants were without a history of speech, language or academic difficulty as per self-report and hearing screened at 25 dB HL at the frequencies 0.5 kHz, 1 kHz, 2 kHz and 4 kHz was normal. All participants were right-handed with the exception of one participant from each language group.

All research methods were carried out in accordance with relevant guidelines and regulations. Experimental protocols were approved by the Internal Review Boards at The Graduate Center, City University of New York, New York, and St. John’s University, Queens, New York. All research participants provided written informed consent in accordance with the established protocols.

### Stimuli

Nonword forms began with the sequences /pət/, /st/ and /sət/, which occur in both the Polish and English languages and /pt/, which is a possible word onset only in Polish. Sound sequences that followed the onsets were matched for rhyme (e.g., /ptɛgɑ/, /pətɛgɑ/, /stɛgɑ/, /sətɛgɑ/). Each onset (/pt/, /pət/, /st/ and /sət/) occurred within 35 different nonwords (e.g., /pt/: /ptilɑ/ /ptugɑ/ /ptɛgɑ/…) and there were two natural productions of each of the 35 nonwords. These 70 productions were presented as first words in the same pairs and the different pairs for a total of 140 presentations for each onset type (e.g., /sət/). The stimuli were produced using a penultimate stress pattern, as evident in Fig. 1 waveforms. The predominant stress pattern in the Polish language is penultimate stress and the English language includes words that have penultimate stress (e.g., petunia).

Stimuli were produced by a bilingual Polish-English male speaker in his mid-twenties. He came to the United States with his family at six years of age and attended school in New York. We identified the speaker of the stimuli as being a dominant English-language speaker. Specifically, an error analyses identified that the speaker reduced the vowel in /pət/, such that native-Polish speakers could not distinguish a small number of nonword productions that began with /pət/ from nonwords that began with /pt/. These nonwords were not included in the current stimulus set. Even though the speaker was dominant in English, the Polish participants, who learned English late in life, judged his Polish pronunciations to be native-like. This is consistent with the speaker’s report that he spoke only Polish in his family home and attended one full day of Polish school each week through 12th grade. Also, we previously demonstrated, and replicated with the current participant groups, that both speakers of Polish and English could differentiate words that began with /st/ versus /sət/, but only native-Polish speakers could distinguish words that began with /pt/ versus /pət/. Further, these behavioral results were confirmed by a late auditory evoked potential (AEP) component of conscious processing^9^.

Five hundred and sixty nonword pairs were randomized and segmented into seven blocks. These seven blocks were presented to the participants in random order. An inter-trial interval (ITI) of 2000 ms separated the word pairs and an inter-stimulus interval (ISI) of 250 ms separated the words within the pairs (Fig. 1). The mean duration (and range) for the nonwords were: /sət/ 698 ms (633–801), /st/ 623 ms (551–728), /pət/ 550 ms (481–671), /pt/ 489 ms (417–604) and the mean duration for the onsets, measured from onset to the burst for /t/, were: /sət/ 258 ms (210–325), /st/ 208 ms (159–259), /pət/ 149 ms (123–174), /pt/ 114 ms (84–140).

### Experimental design

Each of the 48 participants was tested twice, engaging in an attend condition during one experimental session and a passive condition during the alternate experimental session. During the passive condition, participants were instructed to only listen to the stimuli. During the attend condition, participants listened to the nonword pairs and performed a behavioral task to the second word in the word pairs, using a button press response. The participants were asked to determine whether the second word in the nonword pairs had two or three syllables. This task determined whether nonwords containing the onsets /pt/ versus /pət/ and /st/ versus /sət/ could be differentiated by English and Polish listeners.

Attend and passive conditions were counterbalanced such that 12 English and 12 Polish adults engaged in the passive condition during session one and the attend condition during session two. The alternate 12 English and 12 Polish participants engaged in the listening conditions in the reverse order. A minimum of two months (mean 5 months) separated the testing sessions to reduce the effects of stimulus and task repetition^33^.

### Data acquisition

Data was collected at The Graduate Center, City University of New York. The EEGs were recorded using Net Station 4.5.1 (Electrical Geodesics Inc.) in a sound treated, electrically shielded booth, using a 64-channel sensor net. The EEG was collected between 0.1 and 100 Hz with a sampling rate of 500 Hz, and referenced to the vertex electrode Cz. Stimuli were presented free field (62 dB SPL) through left and right diagonally-positioned speakers (Realistic Minimus-7) using Eprime 1.1. EEGs were analyzed using BESA Research 7.1 and BESA Statistics 2.1.

### EEG pre-processing to obtain AEPs

AEPs were used to create a source localization model, which is described below. Bad channels, identified through visual inspection, were interpolated using spherical spline interpolation^34^. Then eye blink and ECG (electrocardiogram) artifact activity were reduced using spatial filtering. Both artifact topographies were modeled by applying principal component analysis (PCA) over averaged blink/ECG events, recognized by a pattern search. For eye blinks, the first PCA component, which explained almost all template variance, was used to reduce eye blink activity. To reduce ECG activity, all components that explained more than 10% of the variance in the averaged template were used. Spatial filtering was performed using the surrogate approach^35^. Data was segmented into epochs of 1500 ms (− 500 pre- to 1000 ms post-stimulus onset). Epochs were scanned for additional artifact. EEG signal within trials that exceeded 120 μV were excluded and EEG signals within trials that contained amplitude jumps (gradient criteria) that exceeded 75 μV were also excluded from further processing. On an individual subject basis, additional channels were interpolated as deemed necessary. In total, an average of 4–5 channels were interpolated (of the 64 total channels) per language group and condition. The average number of accepted trials per word onset type, language group, and condition was as follows: Polish passive: /pt/ 132, /pət/ 132, /st/ 132, /sət/ 131; Polish attend: /pt/ 130, /pət/ 129, /st/ 131, /sət/ 130; English passive: /pt/ 133, /pət/ 133, /st/ 132, /sət/ 132; English attend: /pt/ 128, /pət/ 127, /st/ 129, /sət/ 128. Finally, the averaged data was filtered by 0.1–55 Hz, with a 60 Hz, notch filter.

### Source localization model

A source localization model^36,37^ was applied as a spatial filter, thereby transforming the EEG data into source space (BESA Research 7.1). This process transformed the 64-channel EEG into brain source-level channels, derived through mathematical calculation. Source-level channels were used to enhance efforts to clarify acoustic-level and language-specific processing of phonological sequences through time–frequency analyses.

**Figure 2:**
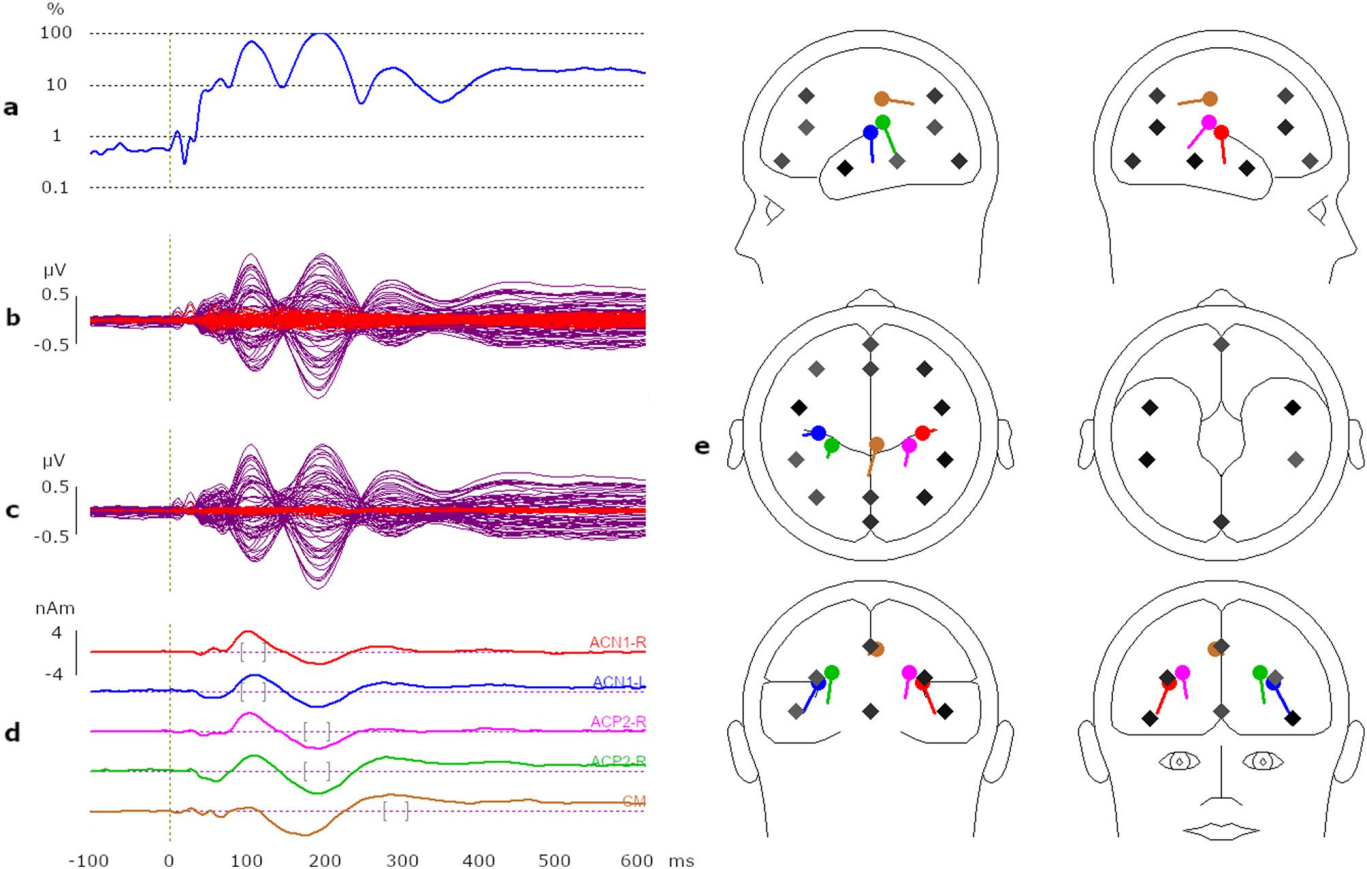
Source Localization Model, Five Dipole + BR regional Sources. (**a**) Illustrates the three GFP peaks consistent with the PCA components in response to the complete data set (**b**) displays the overlay of the five-dipole model (purple) and residual variance (red) and (**c**) illustrates the final five-dipole model + BR regional sources (purple). Notice that the model waveforms (purple) remain similar for both models, but the residual variance (red) is reduced in the final model shown in (**c**). (**d**) Illustrates SWFs obtained at the three-time intervals, 90–120 ms, 170–200 ms and 270–300 ms, consistent with the PCA peaks (**e**) illustrates the five localized source dipoles (red ACN1-R, blue ACN1-L, pink ACP2-R, green ACP2-L) plus the added BR regional sources (BESA 7.1 Research). See text for detail. *GFP* global field power, *PCA* principal component analysis, *SWFs* source waveforms, *BR* brain region.

The source localization model was derived from AEPs, using all participant files in both listening conditions to the phonological-sequence onsets (/pt/, /pət/, /st/, and /sət/) combined in a grand average (~ 53,000 trials) (BESA Research 7.1). A principal component analysis (PCA) identified three peaks of activity, which were consistent with the global field power (GFP) waveforms, shown in Fig. 2a–c. Using an age-appropriate template model (ages 20–24 years)^38,39^, five-dipole sources were localized at the time windows, 90–120 ms, 170–200 ms, and 270–300 ms, which correspond to the timing of the three PCA peaks. The first two peaks coincide with the N1 and P2 components of the AEP. These first two time-windows, 90–120 ms and 170–200 ms, localized bilateral dipole sources in the region of auditory cortex (AC), hence, we use the terms ACN1-R, ACN1-L and ACP2-R, ACP2-L (R = right, L = left) to describe the four source channels. Primary sources for the N1 component originate from the superior temporal plane (STP) (primary auditory cortex, the lateral region of Heschl’s gyrus and planum temporale) with additional smaller contributions from medial-posterior superior temporal gyrus (STG)^40–42^. The second bilateral source identified at the P2 time window, appears posterior to that found at the N1 time window, as evident in the head model, sagittal view of Fig. 2e. Figure 2d illustrates that the N1 and P2 peak activity evident in the source waveforms (SWFs) were only partially separated. This was unavoidable, as the waveforms reflect composite EEG responses to the time-varying acoustic changes within the naturally produced onsets within nonwords. The time window at the third PCA peak (270–300 ms) localized a fifth dipole, a central dipole in the region of the cingulate gyrus (CG) (Fig. 2e). We were confident that the fifth source was central, because attempts to fit bilateral dipoles at this time window resulted in the bilateral dipoles fitting adjacent to a midline location. Orientation for these five dipoles were fitted for each participant’s data in the same time intervals as mentioned above, following the approach described by Hoechstetter et al.^43^. A regularization constant of 1% was used during the entire dipole fitting procedure.

Goodness of fit for the five-dipole model between 50 and 500 ms post-word onset for each of the four onsets, separately, in each language group and listening condition was greater than 92%. To explain possible activity not time-locked to the stimuli, a regional source dipole was added to each brain region (BR), except for those brain regions that were within 3 cm of the modeled five-dipole sources (BESA Research 7.1)^44^. This resulted in a final model containing five-dipole sources and twelve-regional sources. It is worth mentioning that these additional sources do not hamper the five-dipole model because the crosstalk between sources is minimal when a modest regularization of 1% is used. Therefore, sources should contain almost no signal provided that no activity originates from the corresponding brain areas^45^. This approach can be particularly useful for time–frequency analysis, which examines both evoked and induced brain activity. The Talairach coordinates and orientations for all sources are provided in Supplementary materials (Supplementary Table 1). Figure 2c, shows the final model, five dipoles + BR regional sources, and an overlay of residual variance, with all localized sources illustrated in Fig. 2e. Goodness of fit, for the final model (50–500 ms post word-onset) for each of the four onsets, separately, in each language group and listening condition was greater than 98%. Also, a video display (Supplementary Video 1) in supplementary materials illustrates the change in the degree of activity from each dipole in the left and right hemispheres between 0 and 400 ms.

### Source-waveform (SWF) analyses

Using the final source localization model, consisting of five dipoles + BR regional sources, SWFs were created. SWFs are averaged amplitude values, time and phase-locked to the stimulus, from each source-level channel (1–55 Hz; 1500 ms epochs as per AEP pre-processing). SWFs are similar to AEP waveforms, but from source-level, showing the activity of the brain region that is modelled by the source. In the current study, SWF are inverted relative to AEP positive and negative deflections. SWFs were analyzed, in part, to assess the source localization model. For example, to ascertain whether the model’s auditory source-level channels ACN1-R, ACN1-L, ACP2-R, ACP2-L would effectively assess sensory-level processing of the stimuli, we compared the SWFs from all English and Polish participants combined to each onset in the passive compared to the attend conditions. Within-language group comparisons were also conducted for SWFs in response to four onset contrasts /pt-pət/, /st-sət/, /pt-st/, and /pət-sət/.

### Time–frequency (TF) analyses

For T–F analyses, single-trial raw EEG signals were segmented (1500 ms) and transformed into the time–frequency domain using complex demodulation^46^, with 1 Hz wide frequency bins and 50 ms time resolution (BESA Research 7.1). Complex Demodulation is based on the convolution of the EEG signal with series of sine and cosine waves. These calculations are performed in overlapping 50 ms bins, thus, activity is slightly smeared at the edges and may appear to precede the stimulus. A procedure summary can be found in BESA Research resources (https://www.besa.de/wp-content/uploads/2014/05/BESA-Connectivity-1.0-User-Manual.pdf Sect. 8.1, pp. 14). Information pertaining to the width of the time–frequency resolution can be found using the following resource: (http://wiki.besa.de/index.php?title=Time_Frequency_Resolution_In_BESA).

Spectral power and inter-trial phase locking (ITPL) values in response to speech were examined in the LG and theta-frequency bands. Temporal spectral evolution (TSE) values identified the percentage of change in spectral power (amplitude) relative to baseline and reflect both induced (random-phase) and evoked (phase-locked) responses to the stimulus. ITPL values measure the consistency at which the phase aligns to a stimulus across trials within a specific frequency band. A value of 0 indicates random phase alignment and a value of 1 reflects perfect alignment across trials. Language-group effects within LG were reported with the upper limit set at 55 Hz, as a 60 Hz notch filter affected data between 56 and 64 Hz.

As a preliminary test to ascertain whether the model’s auditory source-level channels ACN1-R, ACN1-L, ACP2-R, ACP2-L would effectively assess sensory-level processing of the stimuli, we compared the TSE and ITPL values from all English and Polish participants combined to each onset in the passive compared to the attend conditions. To address the research questions, T-F analyses in response to the phonological sequence onsets /pt/, /pət/, /st/, and /sət/ were conducted on two levels, between-language group and within-language group comparisons.

### Statistical analyses

SWFs and T–F data were analyzed using cluster analysis in combination with permutation testing, designed to minimize the possibility of type 1 error due to multiple comparisons^47–49^ (BESA Statistics 2.1). Initially, preliminary t-tests (two-tailed) comparing data points from two conditions were used to identify clusters of data, showing significant effects that extended across multiple data points. The cluster was formed from neighboring points in the time domain for SWFs or in the time–frequency matrix for T–F data that had p-value lower than the alpha threshold (p < 0.05). A cluster value was then obtained for each identified cluster by summing the t-values for each data point within the cluster. Permutation testing followed, in which the data was randomly re-shuffled 1000 times. For each of 1000 permutations, t-tests were repeated on each data point and new clusters and their values were again obtained. This allowed for a cluster value distribution across permutations. This distribution was used to identify clusters in the data that were non-interchangeable between Polish and English listener groups (between-language group comparisons) or non-interchangeable for contrasting onset sequences (within-language group comparisons) or, non-interchangeable for the passive and attend conditions. To obtain statistical significance we accepted clusters for which more than 950 permutated tests out of 1000 had lower maximum cluster values (*p* < 0.05).

Statistical analyses of SWFs, bandpass filtered between 1 and 55 Hz, were conducted at each time point within the interval 0–400 ms. For statistical analyses of TSE and ITPL values, we assessed a low-frequency range between 2 and 29 Hz and a high-frequency range between 30 and 55 Hz for the time interval 0–400 ms.

### Human subject testing

Experimental protocols were approved by the Internal Review Boards at The Graduate Center, City University of New York, New York, New York, USA and St. John’s University, Queens, New York, USA. All research participants provided written informed consent in accordance with the Institutions’ established protocols.

## Results

### Sensory processing from bilateral auditory source-level channels

Prior to time–frequency analyses, SWFs from the Polish and English participant groups combined in response to each of the four onsets within nonwords were compared in the passive versus the attend conditions. We reasoned that if sensory processing can be determined from bilateral auditory source-level channels, then responses from English and Polish listeners combined should be fairly similar from these source channels for both listening conditions, the passive and attend conditions. Also, within-language group comparisons of SWFs in response to the onset contrasts (/pt/ versus /pət/, /st/ versus /sət/, /pt/ versus /st/, and /pət/ versus /sət/) from bilateral auditory source-level channels should reflect sensory processing differences to the contrasting onsets.

As expected, SWF responses to the speech stimuli were highly similar from bilateral auditory source-level channels in the attend and passive listening conditions. One significant difference was found from an auditory source. From the ACN1-L source a significant difference was found to the /sət/ onset with a larger amplitude for the attend compared to the passive condition at a late time interval (p = 0.015, attend mean 2.306, passive mean 0.49, latency 330–400 ms). This is consistent with our earlier work with this same participant group, in which attention for task (attend condition compared to passive condition) resulted in a negative shift of the AEP waveform, but the AEP waveform morphology that was specific for each onset sequence (/pt/, /pət/, /st/ and /sət/) and reflected sensory processing of the acoustic characteristics of the stimuli remained unchanged^30^.

Within-language group comparisons found significant differences in SWFs to each onset contrast /pt-pət/, /st-sət/, /pt-st/, and /pət-sət/ from auditory source-level channels for each language group and listening condition. This suggests sensory-level processing of the spectro-temporal characteristics within the contrasting onset sequences from bilateral auditory source-level channels (Supplementary Table 2).

For the purpose of ensuring sensory processing from bilateral auditory source-level channels, the same comparisons were conducted on time–frequency data. Comparisons of spectral power and ITPL values in the two-listening conditions from all Polish and English participants combined failed to find significant effects from the model’s sources ACN1-R, ACN1-L, ACP2-R, or ACP1-L. However, numerous significant effects were found when contrasting phonological sequence onsets within each language group, as described in detail below.

### Time–frequency analyses: between-language group comparisons

Between-language group comparisons (Polish, English) of spectral power (TSE) and ITPL values were analyzed in LG and theta to each onset sequence /pt/, /pət/, /st/, and /sət/, individually, from four-dipole sources (ACN1-R, ACN1-L, ACP2-R, ACP2-L) for the passive and attend conditions. Between-language group comparisons found TSE and ITPL values to the onset sequences to be highly similar with one exception, that was in TSE values in LG as described next.

### Spectral power (TSE), low gamma

**Figure 3:**
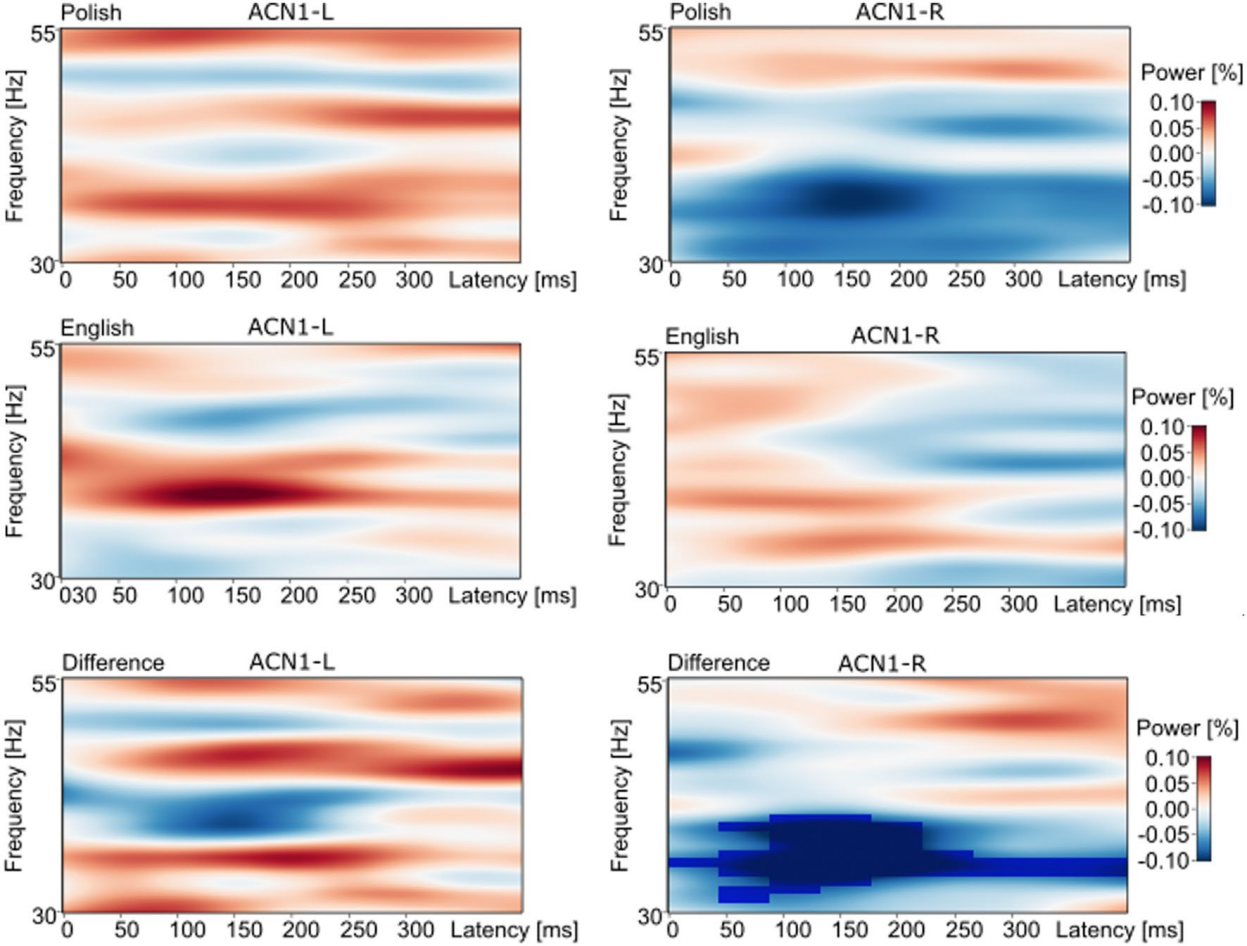
Language-group effects to /pt/ onsets, passive condition. Figure illustrates spectral power (TSE) values for the Polish and English groups from the bilateral auditory source-level channels ACN1-L (left) and ACN1-R (right) in response to the /pt/ onsets, for the passive condition. Significantly larger TSE values were found for the English compared to the Polish listeners to the /pt/ onsets from the right hemisphere source-level channel (ACN1-R) (p = 0.035, 31–40 Hz, 0–400 ms), with blue masking (bottom row, right column) depicting the time–frequency interval for the significant effect. TSE values did not differ for the language groups to the /pt/ onsets from the left hemisphere (p = 0.956).

A significant difference in spectral power was found between the Polish and English listeners in response to the /pt/ onset, the phonological sequence onset that occurs only in the Polish language, from an auditory source-level channel in the right hemisphere (ACN1-R). The values were significantly different in the LG band between 31–40 Hz, 0–400 ms for the passive condition (p = 0.035, Polish mean − 0.072, English mean 0.028). The maximum effect for this response was found at 35 Hz, 150 ms post-onset. Figure 3 illustrates the significant increase in spectral power for the English compared to the Polish listeners, from the ACN1-R source, for an extended time interval and frequency range. For comparison, the figure also shows TSE values from the ACN1-L source, which did not differ between Polish and English listeners for the /pt/ onsets. Additional comparisons of TSE values in LG for the passive condition failed to find significant language group differences to the onset sequences /pət/, /st/ and /sət/, which occur in both the Polish and English languages. Also, no significant differences were found to any of the four onset sequences in the attend condition.

### Spectral power (TSE), theta

No significant language group differences in spectral power were found to any of the four onset sequences in the theta band for either the passive or attend conditions.

### ITPL, low gamma

Language group differences in ITPL values were not found in LG to the four onset sequences for either listening condition.

### ITPL, theta

No significant language group differences in ITPL values were found in the theta band to the four onset sequences for either listening condition.

### Time–frequency analyses: within-language group comparisons

Within-language group comparisons of TSE and ITPL values in response to the onset sequences that differed by a single phoneme (/pt-pət/, /st-sət/, /pt-st/, /pət-sət/) were conducted for the passive and attend conditions. The purpose was to assess differences in sensory processing for each onset contrast and to determine whether sensory processing was comparable across language groups.

### Spectral power (TSE), low gamma

Within-language group comparisons of the onset contrasts from bilateral auditory source-level channels found no significant differences in spectral power in LG for either language group for the passive or attend conditions.

### Spectral power (TSE), theta

**Figure 4:**
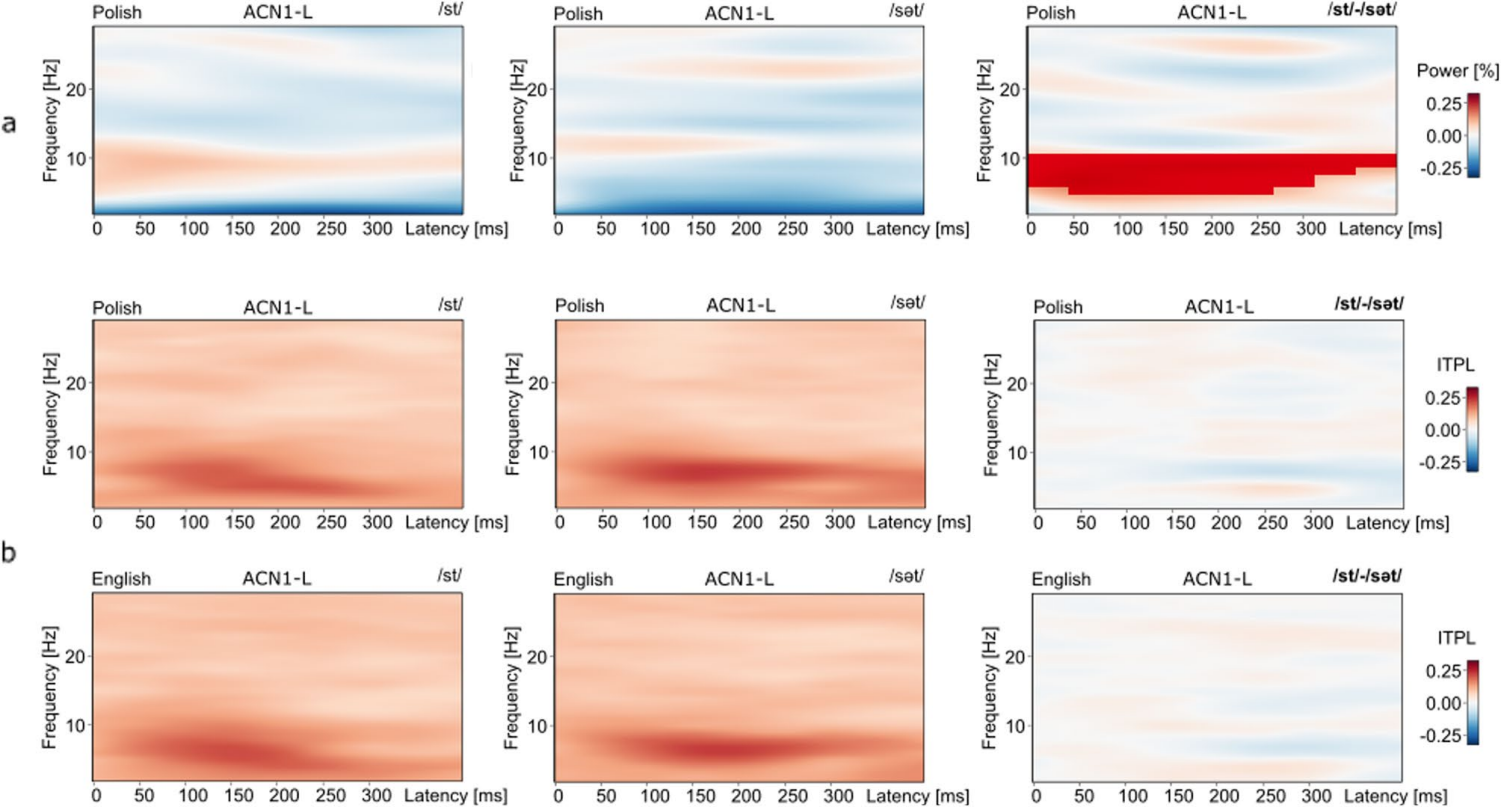
TSE and ITPL effects to the /st-sət/ contrast, attend condition. (**a**) Displays larger spectral power (TSE) in theta to the /st/ compared to the /sət/ onsets for only the Polish group from the ACN1-L source. The mask within the figure on the far right displays the reduced power to the rare /sət/ phonotactic form in the Polish language in high theta (5–10 Hz, max 9 Hz, 0–400 ms, p = 0.009). (**b**) For comparison, significant ITPL effects in theta were not found for either the Polish (top, p = 0.24) or English (bottom, p = 0.61) group from the ACN1-L source in response to the /st-sət/ contrast for the attend condition.

For the attend condition, TSE values in response to the /st-sət/ contrast differed significantly for the Polish group in the high-theta band from the ACN1-L source (p = 0.009, /st/ mean 0.041, /sət/ mean − 0.077, 5–10 Hz, max 9 Hz, 0–400 ms) (Fig. 4). Comparison of TSE values in theta did not find additional significant effects to the contrasts for either language group or listening condition.

### ITPL, low gamma

Within-language group comparisons of the onset contrasts from auditory source-level channels did not reveal significant differences in ITPL values in LG for either language group or listening condition.

### ITPL, theta

Overall, for both language groups, the largest number of significant differences in ITPL values were found in response to the onset contrasts that differed most in spectro-temporal characteristics. That is, a larger number of significant differences in ITPL values were found for the /pt-st/ and /pət-sət/ contrasts as compared to the /pt-pət/ and /st-sət/ contrasts (Table 1a, b). For example, as illustrated in Fig. 1, the /p/ phoneme has low-frequency energy concentration and a short duration, whereas the /s/ phoneme has high-frequency energy concentration and a longer duration of ~ 100 ms compared to the /p/ phoneme. Also, the significant differences in ITPL values to the /pt-st/ and /pət-sət/ onset contrasts were remarkably similar for the Polish and English language groups in time and frequency ranges, as well as in the direction of the mean ITPL values for each onset sequence contrast. Within Table 1, the more positive ITPL mean value for each onset sequence contrast is highlighted in bold text, illustrating consistency in the direction of the mean values for specific time and frequency ranges across language groups. These findings demonstrate replicable patterns of acoustic-level processing to onset sequences in the theta-frequency band across two independent participant groups.

**Table 1:** Within-language group analyses: ITPL values in the theta-frequency band.

Source	Polish participants					English participants					
	p value	Freq	Time	pət mean	set mean	Source	p value	Freq	Time	pət mean	set mean
**a. ITPL Passive Condition /pət/ vs /sət/**											
ACN1-R	0.003	2–8 (6)	0–300	**0.20**	0.14	ACN1-R	0.002	3–12 (6)	0–300	**0.19**	0.13
ACN1-L	0.001	3–7 (5)	0–350	**0.25**	0.16	ACN1-L	0.004	3–8 (6)	0–300	**0.22**	0.15
ACP2-L	0.001	3–10 (5)	0–300	**0.24**	0.16	ACP2-R	0.030	4–6 (4)	50–400	**0.21**	0.15
**a. ITLP Attend Condition /pət/ vs /sət/**											
ACN1-R	0.001	4–11 (5)	0–300	**0.20**	0.14	ACN1-L	0.128 NS	4–6 (4)	0–300	**0.21**	0.16
ACN1-L	0.014	4–7 (5)	0–300	**0.23**	0.15	ACP2-L	0.004	3–9 (5)	0–300	**0.22**	0.16
ACP2-L	0.014	4–6 (5)	0–300	**0.24**	0.16						

Source	p value	Freq	Time	pt mean	st mean	Source	p value	Freq	Time	pt mean	st mean
**b. ITPL Passive Condition /pt/ vs /st/**											
ACN1-R	0.001	5–11 (8)	0–300	**0.22**	0.15	ACN1-L	0.004	5–9 (6)	0–300	**0.23**	0.17
ACN1-L	0.001	4–12 (7)	0–250	**0.24**	0.18	ACN1-R	0.020	2–8 (7)	0–350	**0.19**	0.14
ACP2-R	0.049	5–10 (7)	50–250	**0.22**	0.16	ACP2-R	0.003	5–9 (7)	0–350	**0.19**	0.13
ACP2-L	0.000	4–12 (8)	0–400	**0.23**	0.16	ACP2-L	0.001	5–15 (7)	0–350	**0.19**	0.13
**b. ITLP Attend Condition /pt/ vs /st/**											
ACN1-R	0.001	2–11 (6)	0–300	**0.20**	0.14	ACN1-R	0.005	6–9 (8)	0–300	**0.20**	0.14
ACN1-L	0.000	4–11 (7)	0–300	**0.22**	0.14	ACP2-R	0.002	5–9 (7)	0–300	**0.21**	0.13
ACP2-R	0.002	5–11 (7)	0–350	**0.20**	0.15	ACP2-L	0.001	4–11 (5)	0–300	**0.20**	0.14

Source	p value	Freq	Time	pt mean	pet mean	Source	p value	Freq	Time	pt mean	pet mean
**c. ITPL Passive Condition /pt/ vs /pət/**											
ACP2-L	0.028	3–6 (4)	50–350	0.21	**0.27**						
ACP2-L	0.004	7–10 (8)	0–350	**0.25**	0.19						
ACN1-R	0.006	7–10 (9)	50–350	**0.20**	0.14						
ACN1-L	0.041	8–10 (9)	50–300	**0.22**	0.16						

Source	p value	Freq	Time	st mean	set mean	Source	p value	Freq	Time	st mean	set mean
**d. ITPL Passive Condition /st/ vs /sət/**											
ACP2-L	0.042	6–8 (8)	200–400	0.13	**0.21**	ACP2-L	0.014	6–10 (6)	0–400	0.14	**0.19**
**d. ITPL Attend Condition /st/ vs /sət/**											
						ACP2-R	0.026	6–9 (7)	150–400	0.12	**0.19**
						ACP2-L	0.059	6–8 (7)	150–400	0.13	**0.19**

**Figure 5:**
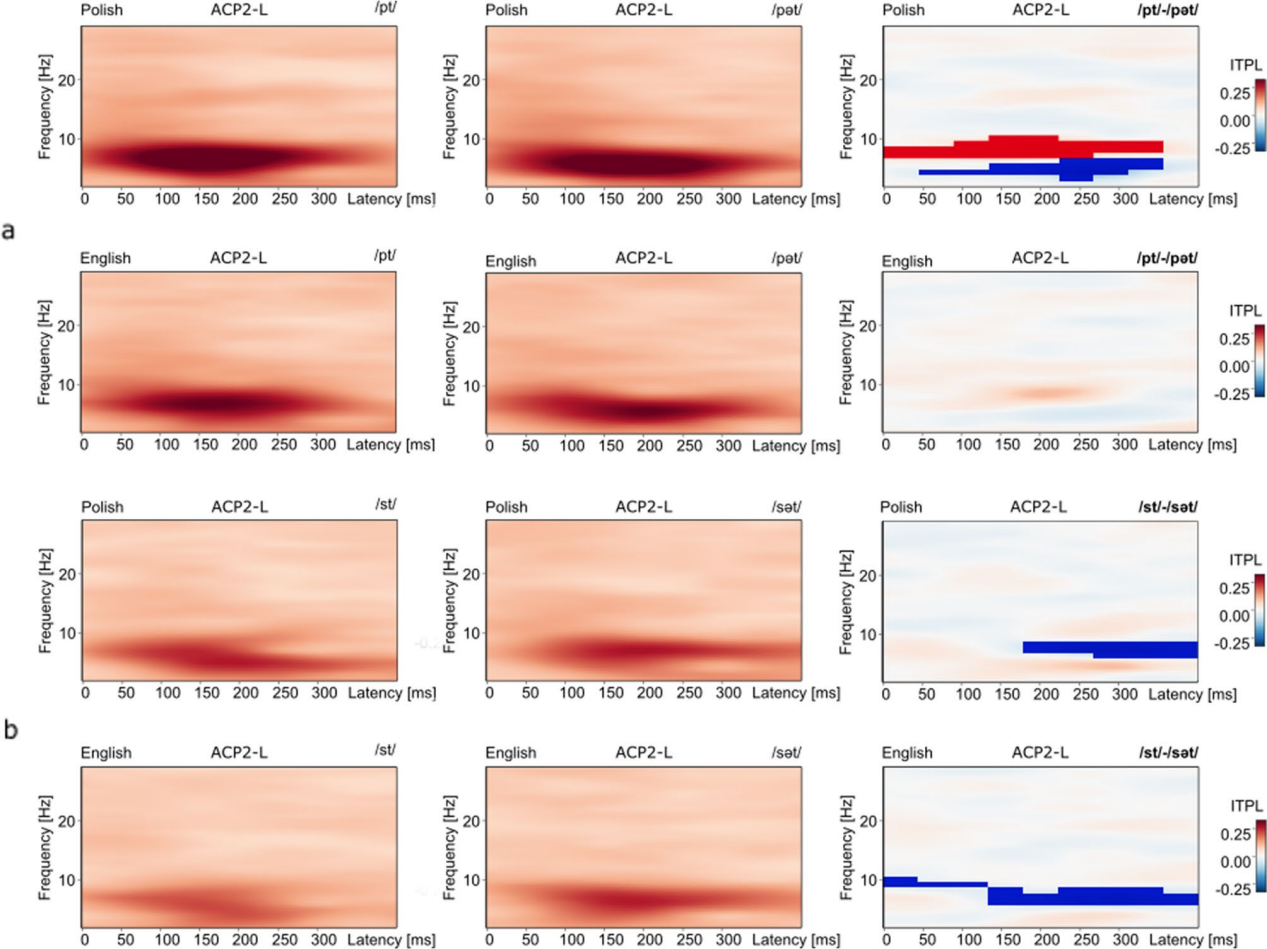
ITPL effects to the /pt-pət/ and /st-sət/ contrasts, passive condition. (**a**) The top row depicts, for the Polish group in the passive condition, significantly different ITPL values to the /pt-pət/ comparison in the high-theta band (max 8 Hz, 0–350 ms, p = 0.004) and the low-theta band (3–6 Hz, max 4 Hz, 50–350 ms, p = 0.028), from the same ACP2-L source. On the far right (top row), red masking depicts larger ITPL values in response to /pt/ in high-theta and blue masking depicts larger ITPL in response to /pət/ in low-theta, reflecting phase locking in select time–frequency bands for different phonological sequences. The bottom row in (**a**) shows a similar ITPL pattern for the English group to each onset sequence, however, differences were not significant. (**b**) Images show the ITPL values to the /st-sət/ comparison from the ACP2-L source, for the Polish (top) and the English (bottom) groups, also for the passive condition. For each group, ITPL values differed, significantly, only in high-theta (Polish: max 8 Hz, 200–400 ms, p = 0.042; English: max 6, 6–10 Hz, 0–400 ms, p = 0.014). Notice in the difference images on the far right, that for both language groups, larger ITPL values in high-theta are in response to the /sət/ onset syllable (blue masking). This is a different pattern than for the /pt-pət/ contrast, which found larger ITPL values in high-theta to the consonant cluster /pt/. See text.

In contrast, whereas acoustic-level processing patterns appeared similar across language groups in response to the /pt-pət/ contrast (Fig. 5a), ITPL values differed significantly only for the Polish group, in the passive condition (Table 1c). No significant differences were found for either group in the attend condition. Also, ITPL values differed significantly for the /st-sət/ contrast for both groups in the passive condition (Fig. 5b), but only for the English group in the attend condition. Table 1d shows that the ITPL effect for the English group in the attend condition to the /st-sət/ contrast was significant from the ACP2-R source-level channel, with a trend from the ACP2-L channel. These ITPL values to the /pt-pət/ and /st-sət/ comparisons, that were contrasted within the experimental design (Fig. 1) may reflect frequency of exposure to the contrasting phonotactic structures in each language, which we discuss below.

Further, Fig. 5 illustrates that the significant differences in ITPL values to the /pt-pət/ comparison, were reflected in dissociated low and high-theta frequency ranges. The dissociated responses in low and high-theta were accompanied by an inversion in the direction of the ITPL mean value, with the larger ITPL values for the /pət/ onsets compared to /pt/ onsets in low-theta and the larger ITPL values to the /pt/ onsets compared to the /pət/ onsets in high-theta (Fig. 5a, Table 1c). This dissociated pattern of response in low and high-theta is visible for both language groups in response to the /pt-pət/ contrast, although, the effect reached significance only for the Polish group. A similar dissociated theta pattern is evident for the /st-sət/ comparison (Fig. 5b). In contrast to this, ITPL values to the /pt-st/ and /pət-sət/ contrasts showed significant differences for particular time intervals across a more-broad theta range.

## Discussion

At cortical levels, acoustic structures associated with linguistic units, including the syllable and phoneme, entrain at oscillatory rates associated with the temporal structure of the linguistic unit, with the rate of acoustic change for the syllable and phoneme tracked in theta and LG frequency bands, respectively^12,13,16,17^. Activations are nested within this hierarchical process, such that oscillations at lower frequencies modulate those at higher frequencies^19–21^. The nature of this integrated, coordinated process is envisioned to support perception.

The current study assessed the association between sensory processing in LG and theta and language-specific perception of nonword onsets from two auditory source-level channels, each in the left and right hemispheres, in native-English and native-Polish speakers. All nonwords are phonotactically legal, and thus possible words in the English and Polish languages, with the exception of nonwords that begin with /pt/, which never occur in the English language. A principal finding of the study was that spectral power differed between the language groups in response to the /pt/ onsets in LG, between 31 and 40 Hz, 0–400 ms from an auditory source-level channel in the right hemisphere (ACN1-R). This singular language-group effect in LG from an early cortical stage of processing, supports the argument that temporal processing facilitates perception^13,20,22,26^. Further, ITPL values in low and high-theta sub-bands that differentiated the acoustic characteristics of the onset sequences /pt-pət/ and /st-sət/ appeared to be modulated by language experience. We discuss these findings below.

### Language-specific processing in LG

We questioned whether language group differences in spectral power or ITPL values in LG would be selective to the /pt/ onset that occurs only in the Polish language and whether the response would be bilateral or restricted to one hemisphere. The English listeners compared to the Polish showed increased spectral power to the /pt/ onsets from the right hemisphere (ACN1-R) for an extended time and frequency interval, without associated differences in the left hemisphere. The Asymmetric Sampling in Time (AST) model purports left-hemispheric asymmetry for rapidly changing phonemic-level signals^13,16,50^, thus, differences in sensory processing for the language groups would be predicted for the left hemisphere. Although counterintuitive, our finding may be consistent with the AST model. The larger power shown by the English listeners to the /pt/ onsets from the right hemisphere source might reflect sensory processing of a novel phonotactic pattern^51,52^. The apparent left asymmetry for the Polish, but not the English listeners, to the /pt/ onsets is consistent with this view. In an earlier AEP study examining these same research participants, we analyzed the P1–N1–P2 complex to the onset sequences from fronto-central electrode sites (0.1–30 Hz) in counterbalanced, attend and passive conditions^30^. Current source density (CSD) maps in that study illustrated that the English compared to the Polish listeners had an increased response to the /pt/ onsets in the passive condition. This AEP effect, which reflects largely phase-locked theta processing was found at fronto-central electrode sites at a late stage of cortical processing between 400 and 900 ms. The current results revealed a similar-response direction with increased sensory processing to the /pt/ onsets for the English compared to the Polish participants for the passive condition, but T–F analyses expanded the previous results by revealing a spectral power effect to /pt/ in LG from the right hemisphere, beginning at an early stage of cortical processing. Sensory processing of syllables has been demonstrated in the left hemisphere auditory cortex in the LG frequency band^22,53^ and it has been argued that activity within LG (28–40 Hz) and theta has a foundation in the evolution of the perception and production of speech^50^. Taken together, the finding of increased power by the English listeners to the unfamiliar /pt/ onset in the right hemisphere might suggest endogenous processing to a novel unfamiliar acoustic–phonetic signal.

Language-specific processing in LG to native and non-native phonological sequences in two-language groups, to the best of our knowledge, has not been previously demonstrated. Therefore, we suggest that presenting an experimental context that highlights phonemic-level contrasts^54^ as well as a passive-listening condition^29,30^ may be beneficial for disentangling automatic patterns of sensory processing in LG, at least for EEG^31,32^.

### Language-specific processing in theta

We then questioned whether language-specific processing would be evident in theta-band activity, which demarcates, primarily, syllabic-level structures^20,21,24,26^. Studies examining language-specific processing have shown mixed effects as to whether theta-band oscillations enhance native-language perception^12,23^. In the present study, ITPL values in theta in response to onset contrasts appeared to reflect acoustic-level and language-specific processing.

ITPL values across single trials in response to the /pt-pət/ onset contrast showed a distinctive pattern in low and high-theta for both Polish and English listeners. However, the differences in ITPL values to the /pt/ versus the /pət/ onsets reached significance only for the Polish listeners in the passive condition (ACP2-L). The /pt-pət/ onset contrast occurs only in the Polish language. The common word “bird”, learned early in life, begins with /pt/ in the Polish language (i.e., “ptak”), whereas the /pVt/ onset is common in word onset in both languages^1–3,55^. In contrast, the /pt/ onset has zero probability of occurrence in the English language^1^. Thus, the ITPL differences in low and high-theta, found only for the Polish listeners, might reflect more variable phase locking by the English listeners to an unfamiliar contrast. Theta oscillatory activity plays a critical role in speech perception with ongoing phase shaping syllable perception^56,57^ and syllable identity^58^. In light of these findings, the suggestion that language input during development might modulate phase synchrony is reasonable.

Further, a language-specific effect in theta is consistent with studies that have examined the T-complex, an AEP component (phase-locked, mostly theta-band activity) from head surface electrodes overlaying lateral posterior temporal brain regions^40^. T-complex modulations have been found to reflect developmental language deficits^59–62^, as well as the amount of acoustic input associated with native-language phonemes and phonotactic patterns^10,63^.

Between language-group analyses failed to find differences in theta to the /pt/ onsets. Acoustic-level processing for the phonological sequences was highly similar across language groups in theta. This may have obscured language-specific effects that may originate from select higher-level brain regions^22,53^.

ITPL values to the /st-sət/ contrast followed a similar pattern of sensory processing across language groups. However, the differences in high-theta to the /st-sət/ contrast reached significance only for the English group in the attend condition from the ACP2-R source, with a trend found from the ACP2-L source. Whereas ITPL values were not significantly different to the /st-sət/ contrast for the Polish group in the attend condition, TSE values from the ACN1-L source to the /st-sət/ contrast were significantly different only for the Polish group. These spectral power effects may reflect cognitive effects of attention, rather than exogenous sensory processing to the onsets, which is evident through replicable ITPL patterns across language groups. We speculate that the combination of responses to the /st-sət/ contrast may reflect the rare occurrence of the /sVt/ onsets in the Polish language^55^. In the English language, both /st/ and /sVt/ are common. Consider the syllable /sVt/ in running speech: phrases such as “set it up”, “set it on”, and “set it out” include the schwa /ə/ within the onset /sVt/ syllable. Our previous neurophysiological studies of Polish and English listeners’ responses to these onsets support this frequency of occurrence data. We have demonstrated a larger late-latency positive component, an AEP component of conscious processing, to the /st-sət/ contrast for the English compared to the Polish listeners^9^, which we have replicated in our lab with this current population of English and Polish listeners. These findings suggest that in addition to the presence (or absence) of a phonotactic pattern in a language, the frequency of occurrence of a phonotactic pattern in a language may modulate sensory processing.

### Acoustic-level processing in low and high-theta, within syllabic-unit tracking

We questioned whether sensory processing of the physical characteristics of phonological-level structures would be evident in LG and/or theta-frequency bands. Temporal processing models predict acoustic structures associated with phonemic units to be tracked in the LG band, however, comparison of onset sequences in each language group (within-language group analyses) failed to find significant spectral power or ITPL effects in LG (between 30 and 55 Hz) from auditory source-level channels. Again, the signals may be small or from select brain regions, and thus undetectable from composite EEG signals.

Peaks in ITPL values within select theta sub-bands differentiated acoustic-level processing to the phonological sequence onsets. This was particularly apparent for the /pt-pət/ and /st-sət/ comparisons. The addition of the vowel, however, did not yield consistent ITPL peaks in either high or low-theta, thus acoustic features of the vowel were not strictly associated with phase-locking values within a particular theta sub-band. Rather, each phonological sequence onset showed a particular ITPL signature pattern that was replicable across language groups, with ITPL peaks selective within theta sub-bands at particular time intervals. The onset consonants /p/ and /s/, within the /pt-st/ and /pət-sət/ comparisons, are highly contrastive in spectral energy. In response to these contrasts, we found theta activation across a broader frequency range, spanning low to high-theta.

At rest, a predominance of low-theta activation (peaking ~ 5.5 Hz) has been localized to the right hemisphere of auditory cortex and a predominance of high-theta activation (peaking ~ 7.5 Hz) has been localized to the left hemisphere of auditory cortex^22^. It was suggested that this asymmetry in theta-band activation might support syllable tracking in the right hemisphere and fine-grain structure monitored for phoneme integration in the left hemisphere. Further, high-theta activity was localized from the superior temporal plane and superior temporal gyrus, purportedly reflecting two stages of processing, which would allow for both phoneme integration and more abstract representation^22,53^. Syllable-level tracking, which has been repeatedly demonstrated in theta, could not be identified in the present study using a match-to-sample experimental design. Rather, our findings revealed acoustic-level processing of structures corresponding to phonological sequences within select time–frequency bands within theta, which may be involved in integration and coordination of phonological information within a hierarchical nested network.

### Attention effect on acoustic-level and language-specific processing

Finally, we examined the effects of attention on acoustic-level and language-specific processing. Spectral power differences in LG and ITPL differences in theta in response to the /pt/ onsets that occurs only in the Polish language, and its contrast with the /pət/ onset, were found during the passive-listening condition. In the attend condition, participants were attending to the stimuli to prepare a button press response to the second word in the nonword pairs.

The language-specific effects found here began at an early stage of cortical processing, an automatic processing stage that is modulated less by selective attention^30,64,65^. This result expands on previous AEP studies that have shown automatic patterns of native-language speech perception to be elicited during tasks for which attention to auditory stimuli was reduced ^29,30^. Automatic processing of lexico-semantic content has also been demonstrated within a non-attend compared to an attend condition^66^. Attention to speech stimuli enhances sensory processing^28,67^, but neural patterns developed in response to speech during critical periods of maturation^68^ may be concealed by additional processing associated with attention and other cognitive demands. Consistent with the model Automatic Selective Perception^31,32^, activation within the attention network may have masked automatic patterns of language-specific processing within the LG and theta-frequency bands.

## Conclusion

In summary, comparison of time–frequency measures in native-English and native-Polish speakers found a single significant effect from auditory source-level channels, a spectral power effect in LG to one of four phonological sequence onsets, the /pt/ onset that occurs in the Polish but not the English language. Phonological sequence onsets were also contrasted in each language group, separately. Whereas patterns of evoked activity were comparable across the language groups, phase-locking appeared modulated by frequency of occurrence of the onset contrasts in the native-language. Our findings, language-specific processing in LG and acoustic-level and language-specific processing in theta are consistent with temporal processing models that posit phonemic-level segmentation in LG, along with coordinated activations between theta and LG support speech perception.

## Supplementary Information

Supplementary Information.

Supplementary Video 1.

## Data Availability

Time–Frequency data used in this study can be found at the Harvard Dataverse Public Repository using the link here: https://doi.org/10.7910/DVN/6SJ1MO.

## References

[1] Vitevitch M, Luce P (2004). A web-based interface to calculate phonological probability for words and nonwords in English. Behav. Res. Methods Instrum. Comput..

[2] Zydorowicz P (2016). Phonotactics and Morphonotactics of Polish and English: Theory, Description, Tools and Applications.

[3] Demenko, G. et al., Development and evaluation of polish speech corpus for unit selection speech synthesis. In *Conference: Proceedings of Interspeech, 2008: 9th Annual Conference of the International Speech Communication Association*, Brisbane, Australia (September 22–26, 2008).

[4] Ortiz-Mantilla S, Hämäläinen J, Realpe-Bonilla T, Benasich AA (2016). Oscillatory dynamics underlying perceptual narrowing of native phoneme mapping from 6 to 12 months of age. J. Neurosci..

[5] Jusczyk P, Friederici A, Wessels J, Svenkerud V, Jusczyk A (1992). Infants sensitivity to the sound patterns of native language words. J. Mem. Lang..

[6] Finney E, Fine I, Dobkins K (2001). Visual stimuli activate auditory cortex in the deaf. Nat. Neurosci..

[7] Näätänen R (1997). Language-specific phoneme representations revealed by electric and magnetic brain responses. Nature.

[8] Dehaene-Lambertz G, Dupoux E, Gout A (2000). Electrophysiological correlates of phonological processing: A cross-linguistic study. J. Cogn. Neurosci..

[9] Wagner M, Shafer VL, Martin BA, Steinschneider M (2012). The phonotactic influence on perception of a consonant cluster /pt/ by native-English and native-Polish listeners: A behavioral and event-related potential (ERP) study. Brain Lang..

[10] Wagner M, Shafer VL, Martin BA, Steinschneider M (2013). The effect of native-language experience on the sensory-obligatory components, the P1N1P2 and the T-complex. Brain Res..

[11] Crone NE, Boatman D, Gordon B, Hao L (2001). Induced electrocorticographic gamma activity during auditory perception. Clin. Neurophysiol..

[12] Ding N, Melloni L, Zhang H, Tian X, Poeppel D (2016). Cortical tracking of hierarchical linguistic structures in connected speech. Nat. Neurosci..

[13] Giraud A, Poeppel D (2012). Cortical oscillations and speech processing: Emerging computational principles and operations. Nat. Neurosci..

[14] Mesgarani N, Cheung C, Johnson K, Chang EF (2014). Phonetic feature encoding in human superior temporal gyrus. Science.

[15] Steinschneider M (2011). Intracranial study of speech-elicited activity on the human posterolateral superior temporal gyrus. Cereb. Cortex..

[16] Poeppel D (2003). The analysis of speech in different temporal integration windows: Cerebral lateralization as ‘asymmetric sampling in time’. Speech Commun..

[17] Ghitza O, Giraud A-L, Poeppel D (2013). Neuronal oscillations and speech perception: Critical-band temporal envelopes are the essence. Front. Hum. Neurosci..

[18] Li Y, Tang C, Lu J, Wu J, Chang EF (2021). Human cortical encoding of pitch in tonal and non-tonal languages. Nat. Commun..

[19] Schroeder C, Lakatos P (2009). The gamma oscillation: Master or slave?. Brain Topogr..

[20] Gross J (2013). Speech rhythms and multiplexed oscillatory sensory coding in the human brain. PLoS Biol..

[21] Lakatos P, Shah A, Knuth KH, Ulbert I, Karmos G (2005). An oscillatory hierarchy controlling neuronal excitability and stimulus processing in the auditory cortex. J. Neurophysiol..

[22] Morillon B, Liégeois-Chauvel C, Arnal LH, Bénar C-G, Giraud A-L (2012). Asymmetric function of theta and gamma activity in syllable processing: An intra-cortical study. Front. Psychol..

[23] Peña M, Melloni L (2012). Brain oscillations during spoken sentence processing. J. Cogn. Neurosci..

[24] Ghitza O (2013). The theta-syllable: A unit of speech information defined by cortical function. Front. Psychol..

[25] Kojima K (2020). Low-frequency neural tracking of natural speech envelope reflects evoked responses to acoustic edges, not oscillatory entrainment. bioRxiv..

[26] Ghitza O (2011). Linking speech perception and neurophysiology: Speech decoding guided by cascaded oscillators locked to the input rhythm. Front. Psychol..

[27] Ghitza O, Greenberg S (2009). On the possible role of brain rhythms in speech perception: Intelligibility of time-compressed speech with periodic and aperiodic insertions of silence. Phonetica.

[28] Lakatos P, Karmos G, Mehta A, Ulbert I, Schroeder CE (2008). Entrainment of neuronal oscillations as a mechanism of attentional selection. Science.

[29] Hisagi M, Shafer VL, Strange W, Sussman E (2010). Perception of a Japanese vowel length contrast by Japanese and American English listeners: Behavioral and electrophysiological measures. Brain Res..

[30] Wagner M (2017). Language experience with a native-language phoneme sequence modulates the effects of attention on cortical sensory processing. Front. Neurosci..

[31] Strange W (2011). Automatic selective perception (ASP) of first and second language speech: A working model. J. Phon..

[32] Strange, W. & Shafer, V. L. Speech perception in second language learners: The re-education of selective perception. In *Phonology and Second Language Acquisition* (eds. Hansen Edwards, J. G. Zampini, M. L.) 153–191 (John Benjamins, 2008).

[33] Nagy ME, Rugg MD (1989). Modulation of event-related potentials by word repetition: The effects of inter-item lag. Psychophysiology.

[34] Perrin F, Pernier J, Bertrand O, Echallier J (1989). Spherical splines for scalp potential and current density mapping. Electroencephal. Clin. Neurophysiol..

[35] Berg P, Scherg M (1994). A multiple source approach to the correction of eye artifacts. Electroencephal. Clin. Neurophysiol..

[36] Scherg, M. Fundamentals of dipole source potential analysis. In *Auditory Evoked Magnetic Fields and Electrical Potentials*, vol. 6, (eds Grandori, F., Hoke, M., Romani, G.-L.) 40–69 (S. Karger, Basel, 1990).

[37] Scherg M, Berg P, Nakasto N, Beniczky S (2019). Taking the EEG back into the brain: The power of multiple discrete sources. Front Neurol..

[38] Richards JE, Xie W (2015). Brains for all the ages: Structural neurodevelopment in infants and children from a life-span perspective. Adv. Child Dev. Behav..

[39] Richards JE, Sanchez C, Phillips-Meek M, Xie W (2015). A database of age-appropriate average MRI templates. Neuroimage.

[40] Howard MA (2000). Auditory cortex on the human posterior superior temporal gyrus. J. Comp. Neurol..

[41] Oganian Y, Chang EF (2019). A speech envelope landmark for syllable encoding in human superior temporal gyrus. Sci Adv..

[42] Steinschneider, M., Liégeois-Chauvel, C. & Brugge, J.F. Auditory evoked potentials and their utility in the assessment of complex sound processing. In *The Auditory Cortex* (eds. Winer, J. & Schreiner, C.) 535–559 (Springer, 2011).

[43] Hoechstetter K (2000). Magnetic source imaging of tactile input shows task-independent attention effects in SII. NeuroReport.

[44] Scherg M (2004). Brain source montages improve the non-invasive diagnosis in epilepsy. Int. Congr. Ser..

[45] Scherg M, Ille N, Bornfleth H, Berg P (2002). Advanced tools for digital EEG review: Virtual source montages, whole-head mapping, correlation, and phase analysis. J. Clin. Neurophysiol..

[46] Papp N, Ktonas P (1977). Critical evaluation of complex demodulation techniques for the quantification of bioelectrical activity. Biomed. Sci. Instrum..

[47] Maris E, Oostenveld R (2007). Nonparametic statistical testing of EEG- and MEG-data. J. Neurosci. Methods..

[48] Bullmore ET (1999). Global, voxel, and cluster tests, by theory and permutation, for a difference between two groups of structural MR images of the brain. IEEE Trans. Med. Imaging..

[49] Ernst MD (2004). Permutation methods: A basis for exact inference. Stat. Sci..

[50] Giraud A-L (2007). Endogenous cortical rhythms determine cerebral specialization for speech perception and production. Neuron.

[51] Posner M, Petersen S (1990). The attention system of the human brain. Annu. Rev. Neurosci..

[52] Sturm W, Willmes K (2001). On the functional neuroanatomy of intrinsic and phasic alertness. Neuroimage.

[53] Giroud J (2020). Asymmetric sampling in human auditory cortex reveals spectral processing hierarchy. PLoS Biol..

[54] Luo H, Poeppel D (2012). Cortical oscillations in auditory perception and speech: Evidence for two temporal windows in human auditory cortex. Front. Psychol..

[55] Dziubalska-Kolaczyk K (2021). Phonotactics and Morphonotactics of Polish and English: Description, Tools and Applications.

[56] Kayser C, Ince RA, Panzeri S (2012). Analysis of slow (theta) oscillations as a potential temporal reference frame for information coding in sensory cortices. PLoS Comput. Biol..

[57] Peelle JE, Davis MH (2012). Neural oscillations carry speech rhythm through to comprehension. Front. Psychol..

[58] ten Oever S, Sack AT (2015). Oscillatory phase shapes syllable perception. PNAS.

[59] Bishop DVM, Hardiman M, Barry J (2012). Auditory deficit as a consequence rather than endophenotype of specific language impairment: electrophysiological evidence. PLoS One.

[60] Bishop DVM, Hardiman M, Uwer R, von Suchodoletz W (2007). Atypical long-latency auditory event-related potentials in a subset of children with specific language impairment. Dev. Sci..

[61] Shafer VL, Schwartz RG, Martin B (2011). Evidence of deficient central speech processing in children with specific language impairment: The T-complex. Clin. Neusophysiol..

[62] Tonnquist-Uhlen I (1996). Topography of auditory evoked long-latency potentials in children with severe language impairment: The P2 and N2 components. Ear Hear..

[63] Rinker T, Shafer V, Kiefer M, Vidal N, Yu Y (2017). T-complex measures in bilingual Spanish-English and Turkish-German children and monolingual peers. PLoS One..

[64] Nourski K, Steinschneider M, Oya H, Kawasaki H, Howard MA (2015). Modulation of response patterns in human auditory cortex during a target detection task: An intracranial electrophysiology study. Int. J. Psychophysiol..

[65] Wöstmann M, Herrmann B, Maess B, Obleser J (2016). Spatiotemporal dynamics of auditory attention synchronize with speech. PNAS.

[66] Shtyrov Y, Kujala T, Pulvermüller F (2010). Interactions between language and attention systems: Early automatic lexical processing?. J. Cogn. Neurosci..

[67] Ding N, Simon J (2012). Emergence of neural encoding of auditory objects while listening to competing speakers. PNAS.

[68] Werker J, Hensch TK (2015). Critical periods in speech perception: New directions. Annu. Rev. Psychol..

