# Supplementary material for: Acoustic-level and language-specific processing of native and non-native phonological sequence onsets in the low gamma and theta-frequency bands
Source: Sci Rep. 2022 Jan 10;12:314. doi: 10.1038/s41598-021-03611-2 (PMC8748887; doi:10.1038/s41598-021-03611-2)
Supplement: Supplementary file 1 — Supplementary Information. [file 41598_2021_3611_MOESM1_ESM.docx]

# Supplementary Materials

Acoustic-Level and Language-Specific Processing of Native and Non-Native Phonological Sequences Onsets in the Low Gamma and Theta-Frequency Bands

Monica Wagner1*, Silvia Ortiz-Mantilla2, Mateusz Rusiniak3, April A. Benasich2`, Valerie L. Shafer4, and Mitchell Steinschneider5

1 St. John’s University, Queens, NY, USA, 11439

2 Rutgers University, Newark, NJ, USA, 07102

3 BESA, GmbH, Gräfelfing, Germany, 82166

4 The Graduate Center of the City University of New York, NY, USA, 10016

5 Albert Einstein College of Medicine, Bronx, NY, USA, 10461

Corresponding Author:

Monica Wagner, PhD

St. John’s University

St. John’s Hall, Room 344 e1 8000 Utopia Parkway Queens, New York 11439 [wagnerm@stjohns.edu](mailto:wagnerm@stjohns.edu)

# Supplementary S-Table 1

**Supplementary S-Table 2 SVideo-1**

**Method: *Source localization model***

S-Table 1 (Supplementary Table 1) contains the Talairach coordinates and orientations for the

five-dipole sources and 12-regional sources located in the final source localization model, Five- dipole + BR regional sources. The five-source dipoles are: auditory cortex N1-right (ACN1-R), auditory cortex N1-left (ACN1-L), auditory cortex P2-right (ACP2-R), auditory cortex P2-left (ACP2-L), cingulate cortex, midline (CM). The regional sources are temporal anterior-left (TAL), temporal posterior-left (TPL), frontal-left (FL), parietal-left (PL), frontal polar-midline (FpM), frontal-midline (FM), parietal-midline (PM), occipital polar-midline (OpM), frontal-right (FR), parietal-right (PR), temporal anterior-right (TAR) temporal posterior-right (TPR).

# S-Table 1

Talairach coordinates and orientations for the five-dipole sources and 12-regional sources


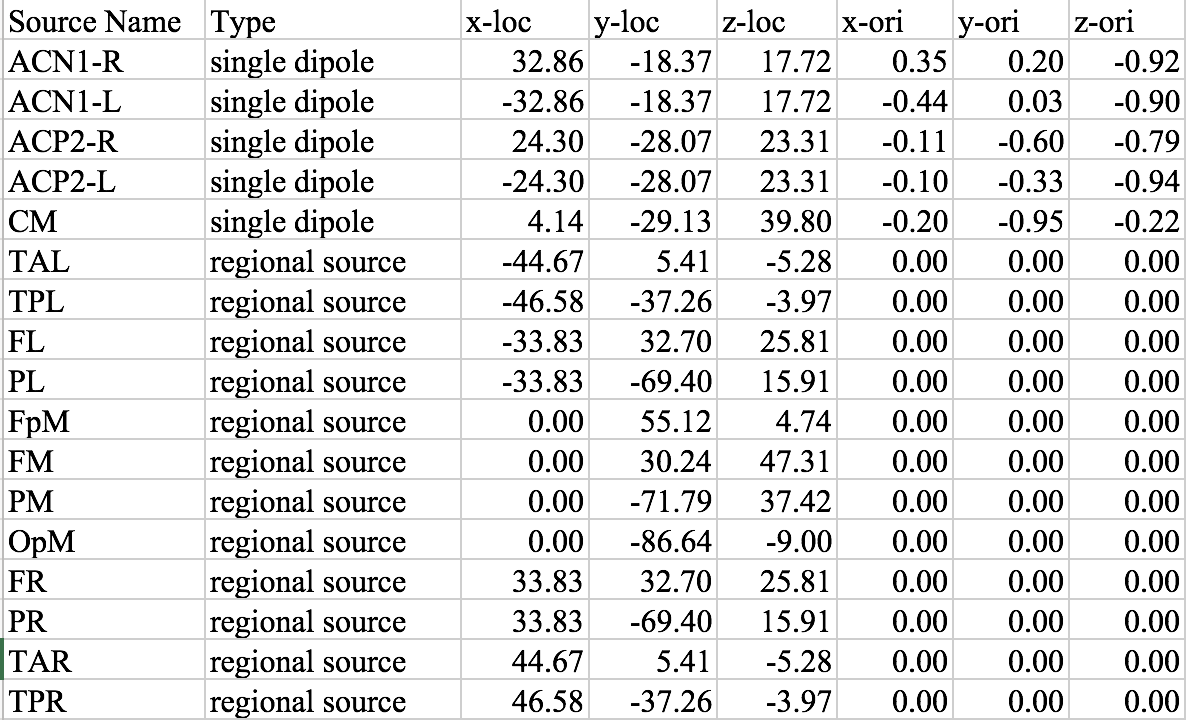


# Results: Sensory processing from bilateral auditory source-level channels

**S-Table 2 (Supplementary Table 2)**

Within-language group effects to contrasting phonological sequence onsets

Source waveforms (SWFs) were compared in response to the four onset contrasts /pt-pət/, /st- sət/, /pt-st/ and /pət-sət/, separately. Each comparison differed by one phoneme. These contrasts were compared within each language group for each listening condition. Significant differences in SWFs were found in response to each contrast in both the English and Polish groups from auditory source-level channels (ACN1-R, ACN1-L, ACP2-R, ACP2-L). This pattern occurred for both the attend and passive conditions. This finding suggests sensory-level processing of the spectro-temporal characteristics that differed for the contrasting onset sequences. Discernable effects of language experience were not found.

# S-Table 2 (Supplementary Table 2)

**SWF Passive Condition /pt/ vs /pət/**

**Polish Participants E nglish Participants**

Source p value

L atency

pt mean pet mean Source p value

L atency

pt mean

pet mean

| ACN1-L | <0. 001 | 194 | 256 | -0. 242 | -2. 594 | ACN1-L | 0. 001 | 238 | 280 | 2. 615 | -0. 18 |
| --- | --- | --- | --- | --- | --- | --- | --- | --- | --- | --- | --- |
| ACP2_L | 0. 013 | 204 | 252 | -0. 246 | -2. 004 | ACN1-R | 0. 009 | 206 | 250 | 0. 086 | -1. 588 |
| ACP2_L | <0. 001 | 268 | 350 | 0. 079 | 2. 93 | ACP2_L | 0. 013 | 288 | 328 | -0. 129 | 2. 428 |
| ACN1-R | 0. 005 | 266 | 316 | 0. 546 | 2. 25 | ACP2-R | 0. 003 | 282 | 328 | -0. 561 | 2. 742 |

**SWF Attend Condition /pt/ vs /pət/**

**Polish Participants E nglish Participants**

Source p value

L atency

pt mean pet mean Source p value

L atency

pt mean

pet mean

| ACN1-L | <0. 001 | 276 | 316 | 1. 147 | 4. 242 | ACP2-R | <0. 001 | 286 | 336 | -0. 732 | 1. 873 |
| --- | --- | --- | --- | --- | --- | --- | --- | --- | --- | --- | --- |
| ACP2-L | 0. 022 | 280 | 306 | 0. 691 | 3. 541 | ACP2-L | 0. 001 | 286 | 340 | 0. 466 | 2. 624 |

**SWF Passive Condition /st/ vs /sət/**

**Polish Participants E nglish Participants**

Source p value

L atency

st mean sət mean Source p value

L atency

st mean

sət mean

| ACN1-L | 0. 001 | 284 | 354 | 2. 134 | -0. 346 | ACN1-L | 0. 028 | 328 | 364 | 1. 882 | -0. 926 |
| --- | --- | --- | --- | --- | --- | --- | --- | --- | --- | --- | --- |
| ACP2-L | 0. 002 | 292 | 352 | 2. 544 | -0. 026 | ACP2-L | 0. 042 | 324 | 358 | 1. 206 | -1. 394 |
| ACN1-R | 0. 016 | 312 | 362 | 1. 503 | -0. 345 |  | | | | | |
| ACP2-R | 0. 04 | 310 | 348 | 1. 894 | 0. 074 |  |  |  |  |  |  |
| ACP2-L | 0. 004 | 194 | 256 | -1. 582 | -0. 089 |  |  |  |  |  |  |
| ACP2-L | 0. 018 | 370 | 400 | 0. 507 | 3. 425 |  |  |  |  |  |  |

**SWF Attend Condition /st/ vs /sət/**

**Polish Participants E nglish Participants**

Source p value

L atency

st mean sət mean Source p value

L atency

st mean

sət mean

| ACP2-R | 0. 011 | 316 | 346 | 2.968 0. 892 | ACP2-R | 0. 011 | 304 | 344 | 0. 721 | -1. 548 |
| --- | --- | --- | --- | --- | --- | --- | --- | --- | --- | --- |
|  |  |  |  |  | ACP2-L | 0. 003 | 316 | 358 | 2. 481 | -0. 555 |

**SWF Passive Condition /pət/ vs /sət/**

**Polish Participants E nglish Participants**

2. 423

| Source | p value | L atency | pət mean | sət mean | Source | p value | L atency | pət mean | sət mean |
| --- | --- | --- | --- | --- | --- | --- | --- | --- | --- |
| ACP2-L | <0. 001 | 262 342 | 3. 104 | 0. 187 | ACP2-L | 0. 003 | 278 | 328 | -0. 744 |
| ACN1-L | 0. 001 | 268 334 | 2. 401 | -0. 449 | ACP2-R | 0. 019 | 284 | 324 | 0. 141 |
| ACN1-L | <0. 001 | 188 252 | -2. 891 | -0. 326 |  | | | | |
| ACP2-L | 0. 001 | 184 242 | -3. 605 | -1. 053 |  |  |  |  |  |
| ACP2-L | 0. 002 | 360 400 | 0. 046 | 3. 062 |  |  |  |  |  |
| ACP2-L | 0. 02 | 76 122 | 3. 788 | 1. 97 |  |  |  |  |  |
| ACN1-L | 0. 022 | 80 134 | 4. 384 | 2. 573 |  |  |  |  |  |

2. 868

**SWF Attend Condition /pət/ vs /sət/**

**Polish Participants E nglish Participants**

Source p value

L atency

pət mean sət mean Source p value

L atency

pət mean

sət mean

| ACP2-L | 0. 002 | 282 | 334 | 2. 96 | 0. 234 | ACP2-L | 0. 001 | 282 | 344 | 2. 547 | -0. 222 |
| --- | --- | --- | --- | --- | --- | --- | --- | --- | --- | --- | --- |
| ACN1-L | 0. 016 | 282 | 312 | 4. 333 | 1. 464 | ACP2-R | 0. 001 | 284 | 340 | 1. 787 | -1. 403 |
| ACP2-L | 0. 043 | 208 | 238 | -3. 316 | -1. 16 | ACP2-L | 0. 032 | 366 | 400 | 0. 206 | 2. 214 |
| ACN1-R | 0. 043 | 366 | 388 | 0. 527 | 3.623 | ACN1-L | 0. 035 | 366 | 400 | 0. 933 | 2. 765 |
| ACP2-L | 0. 003 | 82 | 124 | 2.604 -0. 007 | | ACP2-R | 0. 036 | 366 | 400 | -0. 707 | 1. 944 |
| **SWF Passive Condition /pt/ vs /st/** | | | | | | | | | | | |
| Source | p value | **Polish Participants**  L atency pt mean | | | st mean | Source | **E nglish Participants**  p value L atency | | | pt mean | st mean |
| ACN1-R | 0. 043 | 216 | 256 | 1. 244 | -0. 441 | ACN1-L | 0. 026 | 256 | 296 | 3. 059 | 0. 419 |
| ACP2-L | 0. 022 | 230 | 268 | 1. 929 | -0. 177 | ACP2-L | 0. 027 | 244 | 282 | 2. 178 | -0. 499 |
| ACP2-L | <0. 001 | 292 | 360 | -0. 345 | 2. 477 | ACP2-R | 0. 022 | 308 | 350 | -1. 323 | 1.613 |
| ACN1-R | 0. 006 | 304 | 362 | -0. 467 | 1. 469 |  |  |  |  |  |  |
| ACN1-L | 0. 022 | 298 | 350 | 0. 206 | 2. 408 |  |  |  |  |  |  |
| **SWF Attend Condition /pt/ vs /st/** | | | | | | | | | | | |
| Source | p value | **Polish Participants**  L atency pt mean | | | st mean | Source | **E nglish Participants**  p value L atency | | | pt mean | st mean |
| ACP2-L | 0. 006 | 246 | 282 | 1. 481 | -0. 843 | ACP2-R | 0. 015 86 | | 126 | 2. 416 | -0. 483 |
| ACN1-R | 0. 014 | 244 | 268 | 2. 472 | -0. 215 | ACP2-R | 0. 02 234 | | 278 | 0. 927 | -1. 244 |
| ACP2-R | 0. 022 | 234 | 260 | 1. 881 | -0. 126 | ACP2-L | 0. 002 318 | | 370 | 0. 085 | 2.376 |
| ACP2-R | 0. 002 | 312 | 342 | 0. 449 | 2. 938 |  |  | |  |  |  |
| ACP2-R | 0. 026 | 358 | 380 | 0. 616 | 2. 419 |  |  | |  |  |  |
| ACN1-R | 0. 041 | 336 | 354 | 0. 06 | 2. 521 |  |  | |  |  |  |

# SVideo-1 (Supplementary Video-1)

The video **SVideo-1** illustrates the changes in the degree of predicted neural activity from the five dipole sources between 0-400 ms. The size of the orientation bars in the film depicts the

degree of predicted neural activity that varies in time, by source and hemisphere. The five dipole sources are: red = ACN1-R, blue = ACN1-L, pink = ACP2-R, green = ACP2-L.

Video begins at 0 ms, at word onset, and progresses to 400 ms post-word onset.
